# Non-Cirrhotic Portal Hypertension in Systemic Lupus Erythematosus

**DOI:** 10.7759/cureus.35494

**Published:** 2023-02-26

**Authors:** Silvia Suárez-Díaz, Marta García-Calonge, Guillermo Mendoza-Pacas, Lourdes Mozo-Avellaneda, Luis Caminal-Montero

**Affiliations:** 1 Internal Medicine Department, Hospital Valle del Nalón, Langreo, ESP; 2 Gastroenterology Department, Hospital Universitario Central de Asturias, Oviedo, ESP; 3 Pathology Department, Hospital Universitario Central de Asturias, Oviedo, ESP; 4 Inmunlogy Department, Hospital Universitario Central de Asturias, Oviedo, ESP; 5 Internal Medicine Department, Instituto de Investigación Sanitaria del Principado de Asturias (ISPA) Hospital Universitario Central de ASturias., Oviedo, ESP

**Keywords:** cirrhosis, idiopathic non-cirrhotic portal hypertension, liver involvement, systemic lupus erythematosus, noncirrhotic portal hypertension

## Abstract

We report the case of idiopathic non-cirrhotic portal hypertension associated with systemic lupus erythematosus in a 43-year-old woman who suffered from breast cancer. We review this rare condition, as well as its diagnostic and therapeutic approaches.

## Introduction

Systemic lupus erythematosus (SLE) is a multi-organic autoimmune disease with a wide variety of clinical manifestations. Although any part of the gastrointestinal tract and liver can be involved in SLE, hepatic dysfunction has not been thoroughly investigated. Altered liver function tests in SLE are not uncommon. They are nonspecific, and a complete etiological study should be carried out as in any other patient. Their outcome is usually favorable, and progression to advanced chronic liver disease is rare [1]. Non-cirrhotic portal hypertension (NCPH) is a rare vascular liver disease that is characterized by clinical signs of portal hypertension in the absence of cirrhosis or severe fibrosis. The diagnosis of idiopathic NCPH (INCPH) is always a challenge, as other etiologies must be ruled out. Although it is often not realized, liver biopsy is required for diagnosing this disease [2-3]. NCPH is reported to be associated with autoimmune diseases, but there are only a few cases reported in SLE [4]. Here, we present a case of a woman with SLE and breast cancer complicated by INCPH, which may contribute to further knowledge of this disorder.

## Case presentation

**Figure 1:**
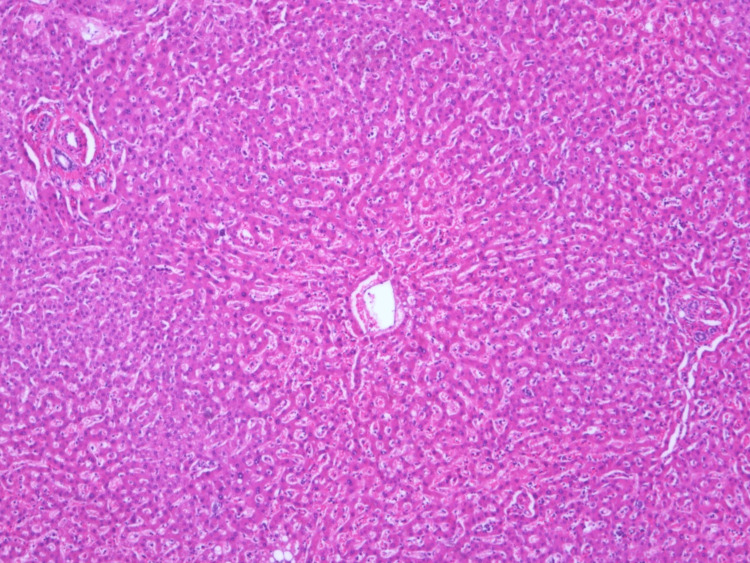
Histological study revealing two portal spaces (ends) and a centroacinar vein, with preserved sinusoidal architecture with mild edema.

**Figure 2:**
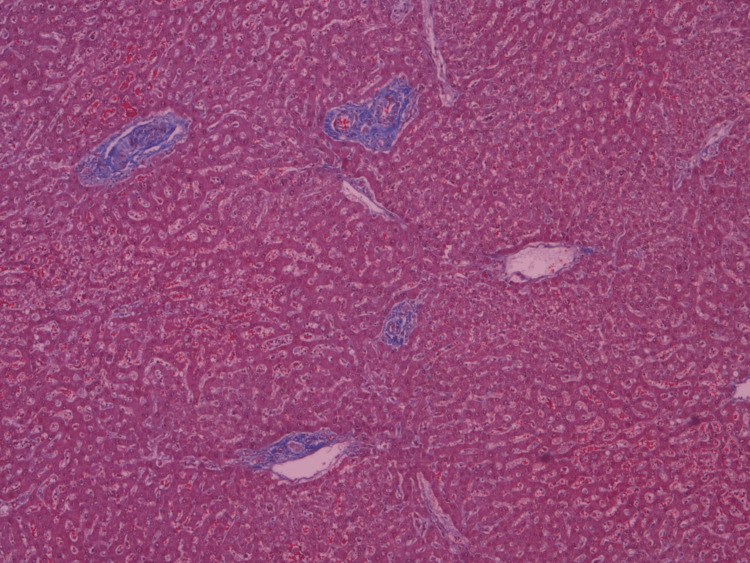
Masson's trichrome stain showing no portal or sinusoidal fibrosis.

A 19-year-old Caucasian female presented in 1997 with discoid facial lupus. Her family history was unremarkable. She rarely consumed alcohol and did not use illicit drugs. She received chloroquine 200 mg once a day and topical steroids during two years, which was discontinued due to improvement and fear of drug toxicity. In 2008, she presented with new facial lesions of lupus discoid and arthritis in wrists and fingers. There was no history of Raynaud’s phenomenon, oral ulcers, or photosensitivity. Immunologic study showed antinuclear antibodies (1:1,280), speckled pattern, double-stranded native DNA, and anti nuclear ribonucleoprotein U1 (anti-RNP), anti-Smith (anti-Sm), anti-Ro/SSA, and anti-La/SSB antibodies were positive with no antiphospholipid antibodies. Peripheral blood examination revealed a mild leukopenia and thrombopenia. Complement levels and urine analysis were normal. Our patient met five criteria of the American College of Rheumatology (ACR) 1982/1987 criteria for SLE. A diagnosis of SLE was made, and she was treated with hydroxychloroquine 200 mg once a day and prednisone 10 mg/day. The prednisone was gradually decreased to a maintenance dose of 5 mg/day. In 2016, she suffered a new SLE flare with arthritis and proteinuria. A renal biopsy was performed, showing mesangial and membranous glomerulonephritis (ISN/RPS [International Society of Nephrology/Renal Pathology Society] class: II+V). She received 2 g of rituximab in one month, prednisone 10 mg/day, hydroxychloroquine 400 mg once a day, rosuvastatin 5 mg/day, and ramipril 2.5 mg/day. One month later, we decreased prednisone to 5 mg/day and hydroxychloroquine to 420 mg once a day. In 2019, she was diagnosed with a stage III infiltrating breast carcinoma. During tumor staging, the abdominal computed tomography (CT) revealed marked collateral vascularization and gastroesophageal varices. Blood liver and coagulation tests were normal. Antinuclear antibodies were positive, and viral serologies were negative. Alpha-1 antitrypsin and ceruloplasmin were normal. Ultrasound imaging showed hepatic steatosis, a permeable splenoportal axis, hypertrophy of the left liver lobe with a slightly lobulated contour, a prominent portal vein, and splenomegaly. Two cords of esophageal varices were found on gastroscopy. Transition elastometry only revealed mild fibrosis (8.8 kPa). She remained without complications of portal hypertension. However, she presented with a progression from her breast tumor despite chemotherapy with cyclophosphamide, liposomal doxorubicin, carboplatin, and paclitaxel, leading to her death in November 2020. Histological study of the liver showed no evidence of portal or sinusoidal fibrosis. (Figures 1, 2).

## Discussion

Liver involvement in patients with connective tissue diseases is not uncommon. Main causes of impaired liver function tests in these patients are drug-induced liver injury, alcohol or metabolic liver disease, and viral or autoimmune hepatitis. Usually, portal hypertension is seen in the context of liver cirrhosis. However, NCPH can also be a complication of different systemic extrahepatic diseases. NCPH is a heterogeneous group of liver disorders that primarily affect the liver vascular system with a portal-caval pressure gradient exceeding 5 mm Hg in the absence of cirrhosis [5-7] (Table 1).

**Table 1:** Disorders and medications associated with non-cirrhotic portal hypertension CREST, calcinosis cutis, Raynaud phenomenon, esophageal dysmotility, sclerodactyly, telangiectasia; HIV, human immunodeficiency virus. VATER, vertebral anomalies, anal atresia, TE fistula (tracheoesophageal fistula) renal defects Adapted from UptoDate

Hematologic/neoplastic	Medications	Inmune	Miscellaneous
Liver cancers	Azathioprine	Rheumatoid Arthritis	Hereditary hemorrhagic telangiectasia
Sacrococcygeal teratoma	Thioguanine	Systemic lupus erythematosus	Turner’s syndrome
Essential thrombocytosis	Trastuzumab	Polymialgia Rheumatica	Cystinosis
Polycythemia vera	Carmustine	Primary biliary cholangitis	VATER syndrome
Myeloproliferative disorders	Arabinoside	Polymyositis	Congenital portal venous anomalies
Lymphoproliferative disorders	Cytosine	Sjögren’s syndrome	Liver transplantation
Multiple myeloma	Busulfan	Scleroderma	Atrial septal defect
Spherocytosis	Doxorubicin	CREST syndrome	Renal transplantation
Sickle cell disease	Cyclophosphamide	Still’s syndrome	Ventricular septal defect
Protein S deficiency	Chlorambucil	Polyarteritis nodosa	Pulmonary vein anomalies
Factor V Leiden mutation	Interleukin-2	Behçet’s syndrome	
Antiphospholipid syndrome	Oxaliplatin	Cryoglobulinemia	
Hyperhomocysteinemia		Idiopathic hypereosinophilic syndrome	
		Celiac disease	
		Myasthenia gravis	
		HIV infection	
		Common variable immunodeficiency	
		Idiopathic thrombocytopenic purpura	

NCPH is usually underdiagnosed because it is asymptomatic. If NCPH is not suspected, portal hypertension signs may be attributed to an unknown liver cirrhosis [6]. Our patient suffered an aggressive breast cancer, but so far there is no relationship in the literature between breast tumors and NCPH, and the liver disease appeared before starting chemotherapy. Abdominal ultrasound with Doppler or CT is required in order to discard portal thrombosis. In NCPH, liver appearance may be normal, or it may show benign regenerative nodules. For definitive diagnosis, a liver biopsy is necessary. Pathological anatomy in NCPH may reveal transformation of the hepatic parenchymal into small, regenerative nodules, but the diagnostic key is showing the absence or minimal fibrosis, unlike cirrhosis. Patients with NCPH have a more benign course than those suffering from portal hypertension due to cirrhosis, due to a preserved liver function. Liver tests are usually normal, and patients may remain asymptomatic. The most common clinical presentation is variceal bleeding, which, in contrast to variceal bleeding in cirrhosis, is relatively well tolerated due to preserved liver function. Splenomegaly is present in more than 95% of patients. Dilated superficial abdominal veins and mild hepatomegaly can also be also seen. Complications such as hepatic encephalopathy, ascites, liver failure, hepatorenal syndrome, and hepatopulmonary syndrome are rare, and they usually appear in late stages of the disease. While data are limited in regard to the best approach, guidelines suggest a similar management of complications as portal hypertension due to cirrhosis. Prophylaxis of complications such as variceal bleeding involves the use of nonselective beta blockers, or band ligation is recommended. SLE complicated by NCPH has been reported previously. Only 25 cases have been described so far [8-12]. The etiology of NCPH in SLE is unknown. Several pathophysiologic mechanisms are believed to be involved, including immunologic alterations and hypercoagulability. Notably, the presence of anti-DNA antibodies has been reported in up to 65% of Japanese patients with INCPH [10]. As autoimmune factors are considered to underlie the development of SLE-associated NCPH, some authors suggested that steroid could be a therapeutic option [8], but there is not recommendation on the use of immunosuppressants.

## Conclusions

In conclusion, although NCPH in association with SLE is rare, it should be considered in any patient suffering from clinical manifestations of portal hypertension in the absence of cirrhosis. A diagnosis of suspected NCPH is critical to avoid diagnostic delays, which will lead to improved treatment options and prognosis.
